# Design and Development of Photonic Biosensors for Swine Viral Diseases Detection

**DOI:** 10.3390/s19183985

**Published:** 2019-09-15

**Authors:** Amadeu Griol, Sergio Peransi, Manuel Rodrigo, Juan Hurtado, Laurent Bellieres, Teodora Ivanova, David Zurita, Carles Sánchez, Sara Recuero, Alejandro Hernández, Santiago Simón, Gyula Balka, Ioannis Bossis, Alessandro Capo, Alessandra Camarca, Sabato D’Auria, Antonio Varriale, Alessandro Giusti

**Affiliations:** 1 Universitat Politècnica de València Nanophotonics Technology Center, 46022 València, Spain; juahurmo@ntc.upv.es (J.H.); blaurent@ntc.upv.es (L.B.); tivanova@ntc.upv.es (T.I.); dazuher@ntc.upv.es (D.Z.); 2 Lumensia Sensors S.L., 46022 València, Spain; speransi@lumensia.com (S.P.); mrodrigo@lumensia.com (M.R.); csanchez@lumensia.com (C.S.); srecuero@lumensia.com (S.R.); ahernandez@lumensia.com (A.H.); ssimon@lumensia.com (S.S.); 3 University of Veterinary Medicine, 1078 Budapest, Hungary; balka.gyula@univet.hu; 4 Agricultural University of Athens, 11855 Athens, Greece; bossisi@aua.gr; 5 Institute of Food Science, CNR, 83100 Avelino, Italy; alessandro.capo@isa.cnr.it (A.C.); alessandra.camarca@isa.cnr.it (A.C.); sabato.dauria@cnr.it (S.D.); antonio.varriale@isa.cnr.it (A.V.); 6 CyRIC, Cyprus Research and Innovation Centre, Nicosia 2414, Cyprus; alessandro@cyric.eu

**Keywords:** swine disease, photonics, antibody, ring resonator, photonic integrated circuit (PIC)

## Abstract

In this paper we introduce a field diagnostic device based on the combination of advanced bio-sensing and photonics technologies, to tackle emerging and endemic viruses causing swine epidemics, and consequently significant economic damage in farms. The device is based on the use of microring resonators fabricated in silicon nitride with CMOS compatible techniques. In the paper, the designed and fabricated photonic integrated circuit (PIC) sensors are presented and characterized, showing an optimized performance in terms of optical losses (30 dB per ring) and extinction ration for ring resonances (15 dB). Furthermore, the results of an experiment for porcine circovirus 2 (PCV2) detection by using the developed biosensors are presented. Positive detection for different virus concentrations has been obtained. The device is currently under development in the framework of the EU Commission co-funded project SWINOSTICS.

## 1. Introduction

Traditional sensor devices are designed to detect changes in terms of physical or chemical parameter modifications. These sensors are based on a high number of detectable parameters (conductivity, mass, optical properties, etc.) and present a lot of potential applications [1,2].

In recent years, there has been great interest in the scientific and industrial world on the research and development of optical sensors for detection of changes in several environmental magnitudes that allow detection of dangerous gases, explosives, UV radiation, vibrations, and color changes [3,4,5,6,7,8]. Moreover, optical sensors have also been proposed to be used in medical and bio applications, detection of viruses and bacteria, and even for food and health quality detection [9,10,11,12,13,14,15].

The first advantage of using an optical sensor opposed to traditional electrical based sensors is that electrical sources are not required. In addition, optical sensors can operate at environmental temperatures, which means that an optical sensor can be used in critical environments and with dangerous products (explosives, chemical products, etc.), where the use of sensors based on conductivity changes is not allowed. Furthermore, optical sensors present better performance in terms of interference problems, and allow a detection level beyond the state of the art. Moreover, optical sensors can be used for multiple analytes detection, giving more complete and useful information to final users.

On the other hand, the development of new micro and nano instruments and technologies, and their heterogeneous integration into smart systems, has become one of the key enablers in achieving the next generation of advanced photonic biosensors, which will be used in real and high impact scenarios. To achieve this goal, the next generation of photonic biosensors must be based on systemic miniaturization and integration of heterogeneous technologies, functions, and materials, and must ensure the convergence of nano, bio, and information and communications technologies (ICT) to achieve smaller, smarter, and energy autonomous devices and systems. In addition, this heterogeneous integration (photonic/bio and new materials) is also a key factor in reaching miniaturized and multisensing photonic integrated circuits (PIC) and systems able to sense, understand context, and communicate. Having these fast, efficient, and reliable multisensing devices and systems is essential in many fields such as medical diagnostics, food safety control, or environmental control. In addition using nanostructures allows for high interaction with the matter and faster responses.

In this paper, the development and test of bio photonic sensors carried out in the frame of the SWINOSTICS (swine diseases field diagnostics toolbox) project [16,17] have been tackled, considering the abovementioned advanced bio-photonic sensor requirements. In this sense, the SWINOSTICS approach is based on the combination of photonic integrated circuit (PIC) technology and bio-sensing technology to detect different swine viruses (each virus presence will be detected by one of the functionalized resonant rings forming the PIC). The aim of the present paper is to describe the design and fabrication of the biosensor photonic integrated circuit (PIC) which is, as mentioned, based on resonant rings fabricated in silicon nitride technology as will be described in Section 2.

As mentioned, the SWINOSTICS diagnostic bio sensors and tools will be used in a strategic field such as livestock products, which have dramatically increased in demand during the last 40 years due to the increasing human population, urbanization rates, and income growth rate. Concretely, SWINOSTICS targets six emerging and endemic swine viruses: African swine fever (ASF) [18], classical swine fever (CSF) [19], porcine reproductive and respiratory syndrome (PPRS) [20], porcine parvovirus (PPV) [21], porcine circovirus 2 (PCV2) [22], and Swine Influenza A (SIV) [23]. Traditionally, these virus detections in the laboratory rely on two different approaches: direct detection of the pathogen by polymerase chain reaction (PCR), enzyme-linked immunosorbent assay (ELISA), or cell culture-based virus isolation, and/or detection of circulatory antibodies in blood samples. All of these are expensive, require trained personnel, and are not properly suitable for a portable field device as proposed in the SWINOSTICS project. The use of the proposed biosensors will allow an important reduction in the detection time and cost in such a high impact field as livestock.

In the present work, a concrete experiment leading to the detection of Porcine Circovirus 2 (PCV2) using the proposed and developed biosensors will be proposed and demonstrated. The results of PCV2 detection for different concentrations are shown and described in Section 3 of the paper.

## 2. Sensor PIC Design and Fabrication

The proposed biosensors are based on a photonic transducer, which uses resonant cavities; concretely ring resonators fabricated in silicon nitride technology. The use of photonic high-quality factor ring resonators has been widely proposed for many applications, the development of sensitive refractive index sensors being one such application. Biological sensor arrays based on high quality factor evanescent microring waveguide resonators have been demonstrated and fabricated with technical processes which are fully compatible with CMOS fabrication [24,25,26,27,28,29,30,31]. In addition, the use of silicon nitride technology offers several advantages compared to technologies, such as silicon on insulator (SOI), as there are more robust structures against fabrication tolerances and enhanced interaction for sensing applications [27].

The biosensors PICs proposed in this paper were fabricated on silicon nitride 6” wafers, comprised of a top layer of 300 nm of silicon nitride deposited by LPCVD (low chemical vapor deposition) on a 3.26 umthick layer of thermal silicon dioxide to achieve low coupling loss gratings [31]. In order to ensure monomode propagation, the width of the silicon nitride waveguides was fixed to 1.1 microns.

**Figure 1:**
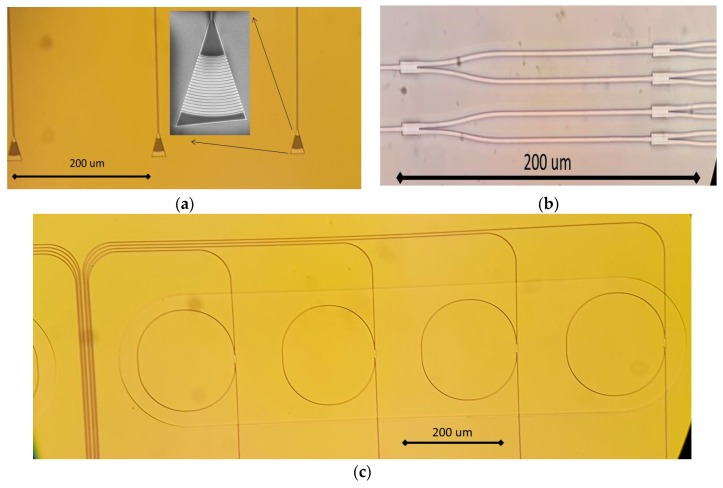
Microscope images of the fabricated biosensor Photonic Integrated Circuit (PIC) building blocks; (**a**) grating coupler, (**b**) power splitting block, and (**c**) ring resonators.

Our biosensor PIC comprises three main building blocks: sensing ring resonators, light coupling block (grating couplers), and optical power distribution block. The ring resonators are used as sensor elements (using one resonant ring for each targeted virus), the light coupling section allows to introduce and extract the optical signals into/out of the PIC whilst the power distribution block is necessary to feed all the resonator rings of the PIC with a common laser source input. After preliminary simulations, and in order to optimize each building block and validate the fabrication process, a first run of each functional block was fabricated sweeping different physical parameters to determine and fine-tune the most suitable physical structures. This optimization was performed searching for the best PIC overall performance in terms of sensitivity, which is related directly with sensing parameters as limit of detection (LoD), limit of quantification, etc. Figure 1 shows some optical microscope images of the fabricated building block structures (gratings, splitters, and rings).

After characterizing optically the fabricated building blocks at lab, the more convenient functional elements were selected and included in the complete biosensor photonic integrated circuit (PIC) design. In this sense, the selected resonator rings showed sensitivities around 265 nm/RIU with quality factors around 25,000, which are high enough for our sensing applications. In addition, and based on our system noise (1.89 pm) and temperature drift, we calculated a detection limit of 7.13 × 10^−6^ RIU, which is in the range of the best values obtained for planar ring resonators [26,27,28]. Moreover, the gratings couplers showed coupling losses of ~7.5 dB and the distribution elements based on 1 × 2 MMI’s showed splitting losses of ~4.2 dB. 

**Figure 2:**
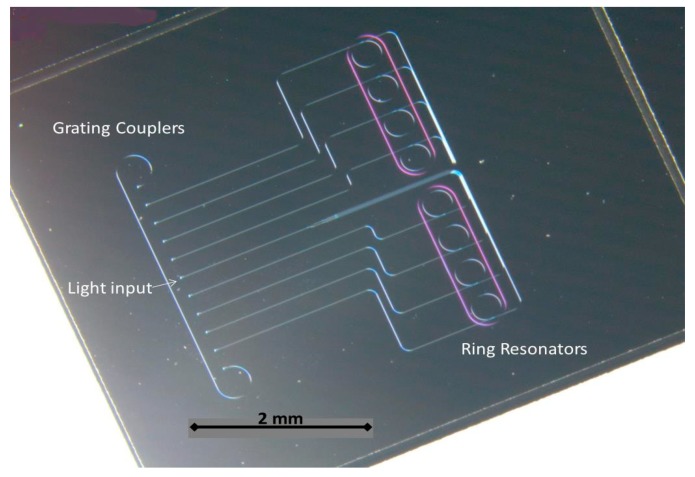
Real fabricated biosensor PIC (image obtained by optical microscope).

Once the basic structures were optimized, the complete design of the PIC was transferred to the silicon nitride samples and wafers, as shown in Figure 2, by using a CMOS compatible fabrication process carried out in a class 10–100 cleanroom environment. Our fabrication process is based on a direct writing electron beam process on a poly-methyl methacrylate (PMMA) positive resist layer with a thickness of 100 nm. After developing the resist, a metal mask is created by chromium evaporation prior to a lift-off process. This metal mask was used to perform the inductively coupled plasma-reactive ion etching (ICP-RIE) of the silicon nitride layer using fluoride gases. With the mentioned process, we were capable of obtaining a good performance in terms of roughness and side wall verticality in the final fabricated samples (propagation losses ~2.5 dB/cm). Finally, after depositing a silicon dioxide layer (one micron thick) by plasma enhanced chemical vapor deposition (PECVD), a window was opened on the resonant rings by removing the silicon dioxide with a second e-beam lithography and a new ICP-RIE etching. Figure 2 shows a real image of the fabricated biosensor, obtained by means of an optical microscope Leika M205C).

As can be seen in the above image, the fabricated biosensor PIC has eight resonant photonic rings. Six of them will be functionalized with specific antibodies to detect the targeted viruses, whilst the other two rings will be used to perform positive and negative control during the sensing process. The opened channels on the rings can be also observed in Figure 2, as well as the grating couplers on the right side of the PIC.

**Figure 3:**
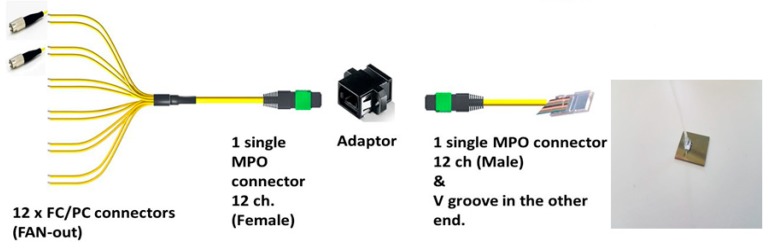
Fiber array attachment and optical connectors.

To finish the fabrication process, and in order to allow the injection of light into the PICs and extract it from the rings to perform sensing, a fiber array (FA) was attached to the biosensor PIC. This attachment process was carried out in an alignment bench which allows the right alignment and attachment of the FA on the PIC grating couplers. Figure 3 depicts the used fiber optics, which allows the PIC to connect to the laser and the photodiodes to perform the sensing. In our design, fiber array with a Multi-fiber Push On (MPO) connector was attached to the PIC. The use of such MPO connector makes easier the exchange of the PIC, since only one connector has to be removed. The FA will be connected to a fan-out element with 12 fibers, one corresponding to the laser signal, 8 for the ring optical outputs, and 3 back-up fibers.

**Figure 4:**
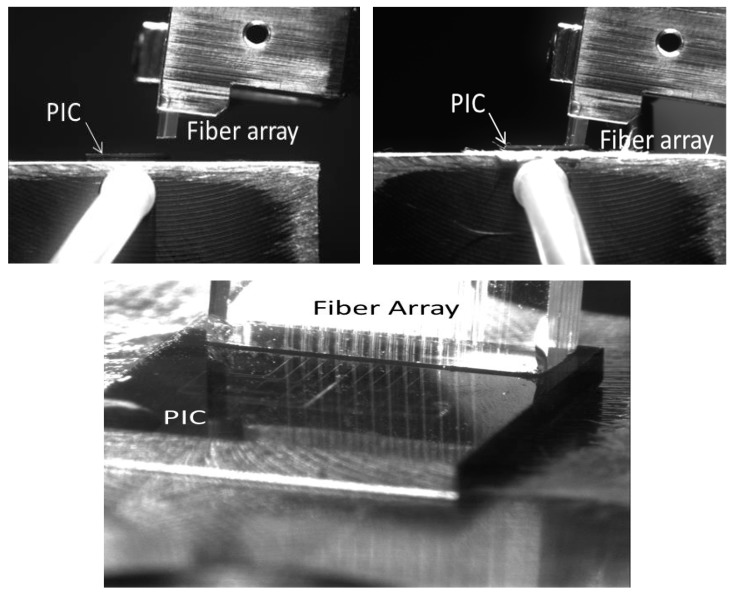
Biosensor fiber array attachment process.

As mentioned, using an alignment bench (concretely a MICOS system alignment bench with 16 alignment axes), the attachment of the fibers was successfully reached following the process depicted in Figure 4. A dynamic process consisting of measuring the optical signal through a PIC reference waveguide was used to optimize the FA placement, just on the gratings. Once the maximum power was reached after several iterations, the FA was glued to the PIC with a resist (concretely Dymax OP52 optical adhesive) cured by UV light.

## 3. Optical Sensing Results

Once the photonic sensor PICs were fabricated, different optical characterizations were performed. Fabricated PICs before surface functionalization were optically characterized in the same alignment bench in order to test the ring performance.

In a second step, a first characterization of functionalized PICs was carried out at lab level with real swine PCV2 virus samples.

### 3.1. Non-functionalized PICs Optical Characterization

The sensor PICs were characterized just after the fiber array attachment. This first characterization was performed on non-functionalized chips, just to verify the optical response of the resonator rings in comparison with expected results from simulations and parameter optimization. 

**Figure 5:**
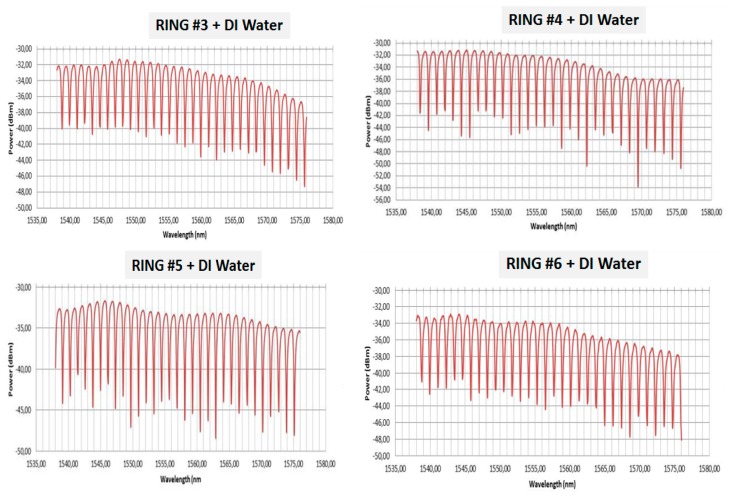
Optical response of several ring resonators of a fabricated biosensor PIC.

This preliminary characterization was performed after gluing the fiber array (following the process showed in Figure 4) and dropping deionized water (DI water) on the rings to emulate the real liquid sample flowing. Figure 5 shows the optical response for four resonant rings of one of the fabricated sensor PICs (rings #3 to #6 corresponding to the 4 rings in the middle area in Figure 2). 

As observed in Figure 5, the fabricated biosensor PICs show a typical microring resonator response where the corresponding resonant notches can be observed. The sensing principle is based on following the position of a selected notch, whilst flowing the sample collected from the animals. In case of a positive detection of a targeted virus, a shift in the notch placement will be observed during the measurement. As mentioned, each ring will be functionalized with a specific antibody capable of detecting the corresponding virus. As expected in simulation and design stages, and after building blocks fabrication and optimization, the microring resonators show optical power losses around 30 dB, and extinction ratios around 15 dB, which can also be observed in Figure 5.

### 3.2. Functionalized PICs Optical Characterization

Several photonic sensors PICs were functionalized with the aim of detecting PCV2 virus. To carry on this functionalization, the surface of the biosensor PIC was firstly oxidized. Then, the surface was rinsed with deionized water (DIW) and dried. After that, the silanization of the surface was carried out with a 3-chloropropyltriethoxysilane (CTES) solution. The activation of the group carboxylic from the CTES organosilane over the surface of the biosensor PIC was carried out by the addition of a mix of carbodiimide and N-hydroxysuccinimide (EDC/NHS). The incubation was done at room temperature for 30 min and then the surface was rinsed and dried with air flow. On top of the surface, commercial rabbit polyclonal antibodies against PCV2 virus (*Thermo Fisher Scientific #PA5-34969*) were covalently immobilized in an oriented manner. The biosensor PIC was incubated in an atmosphere with controlled humidity for 2 h, then the surface was rinsed with Phosphate buffered saline (PBS) and dried again.

**Figure 6:**
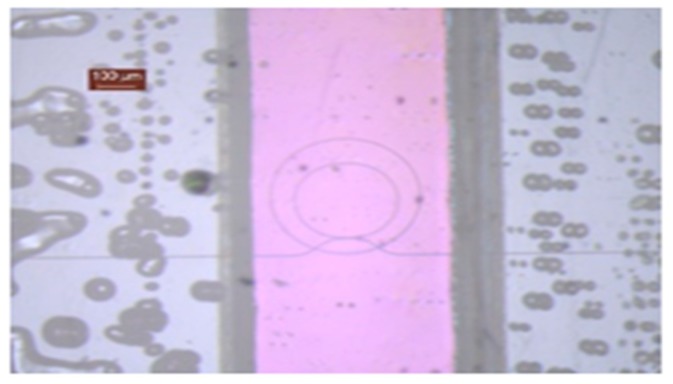
Fluidics detail and interconnection holder.

Once the photonic chips were functionalized, it was necessary to attach a microfluidic adhesive layer that allowed the flown of the sample. For this experiment, an available simpler microfluidic layer (not the final microfluidics design to be used in SWINOSTICS project) was bonded on the chip surfaces. Figure 6 depicts the attached microfluidics layer where it can be observed as the microfluidic channel was aligned on top the sensor resonant microrings.

The prepared photonic chips were used for virus sensing by using an optimized laboratory set-up which makes use of a peristaltic pump. To perform the PCV2 sensing experiment, a flowing protocol was designed. First, a phosphate buffered saline with Tween-20 (PBST) solution was flowed for 1–2 min to get a reference signal. After that, the virus sample diluted in the same buffer was flown for 10 min, and finally a cleaning buffer (PBST as in the first step) was flown for 1–2 min. In the experiment, the initial virus sample was a real sample with unknown concentration, and all the dilutions were prepared from this starting virus sample. So, to perform the experiment, several PCV2 virus samples were flowed on functionalized ring resonators at several dilution factors, from a 1/100 to 1/5000 dilution in PBS buffer.

**Figure 7:**
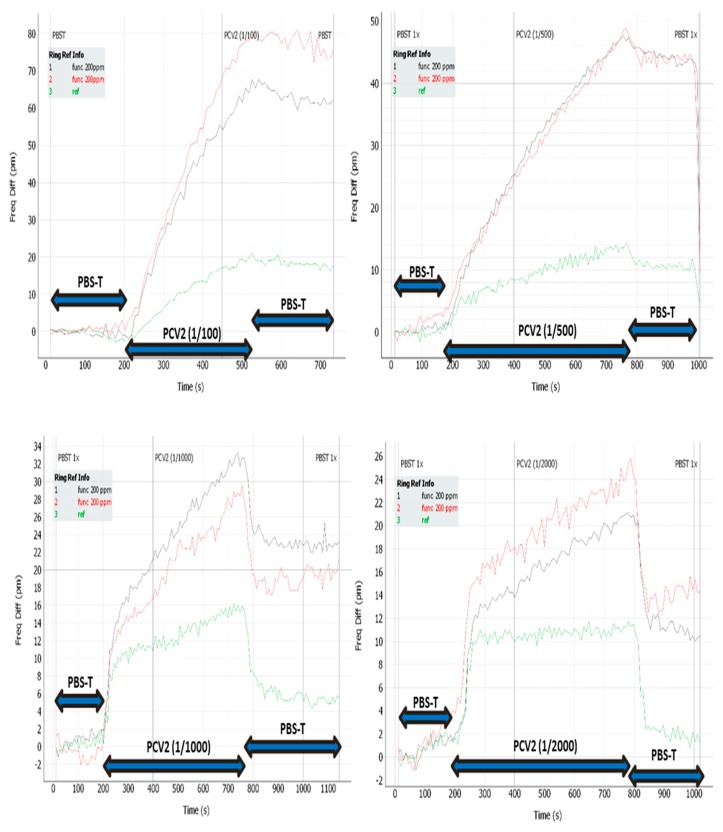
Optical measurements with different PCV2 virus concentrations.

Figure 7 shows the plots of a PCV2 sensing experiment where rabbit polyclonal antibodies were immobilized on two of the ring resonators (red and black lines) whilst another not functionalized ring (green line) was used as a reference. In Figure 7, the resonant notch shift of the three rings are plotted during the overall flowing process for four different concentrations of PCV2 viruses obtained from the same starting sample (1/100, 1/500, 1/1000 and 1/2000). The starting sample corresponds to a real sick animal with high/moderate amount of PCV2 virus concentration. 

As it can be observed in the plots in Figure 7, the two ring resonators which were functionalized with polyclonal antibodies, responded positively against the virus, as a remarkable shift in the resonance notch of the microring is observed. In contrast, the microring resonator which was not functionalized (reference ring) just showed minor changes in its resonance position due to the unspecific binding which occurs in this case. 

As observed in Figure 7, during the first flowing of PBST solution, the resonance is practically not shifted. After that, the virus sample diluted in the same buffer flowing produces a shift which is dependent on the dilution. Finally, the cleaning buffer flowing helps to eliminate unspecific bindings (which seem to be higher for higher dilutions). The sensing principle consists in comparing the resonance shift of the functionalized ring with the reference (non-functionalized) ring.

**Figure 8:**
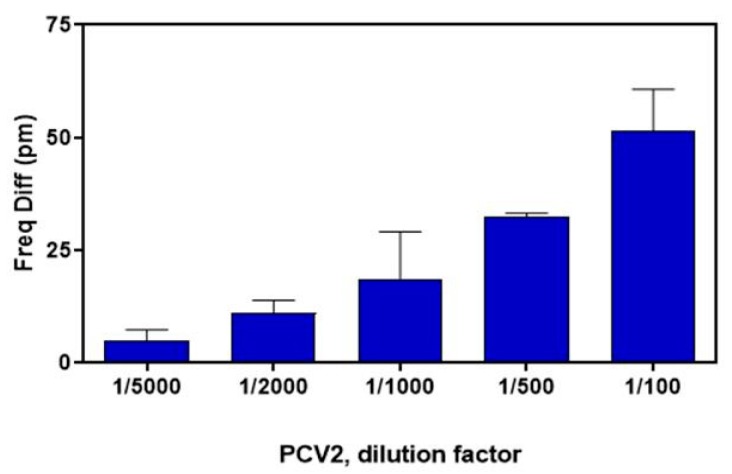
Response against PCV2 virus at different dilution factors (from 1/100 to 1/5000 in PBS buffer).

Finally, in Figure 8 the photonic measurement responses (in terms of microring resonance notch shift) obtained during the experiment are shown against PCV2 virus for different dilution factors (from 1/100 to 1/5000 in PBS buffer). As it can be seen, positive detection was achieved up to 1/5000 dilution factors. It can be also observed that there is a clear dependence on the achieved results as a function of the virus concentration. So, for the more diluted sample, the less intensive optical signal is obtained.

## 4. Discussion and Conclusions

In this paper, the design and fabrication of photonic biosensors based on microrings has been presented. The biosensors were fabricated in silicon nitride by using CMOS compatible techniques as electron beam lithography and RIE-ICP etching. The biosensor proposed is comprised of eight resonant rings, which will be functionalized with different antibodies to detect specific virus affecting swine livestock in the frame of the H2020 SWINOSTICS project. The fabricated microrings have been statically characterized after the fiber attachment, obtaining a good performance in terms of optical losses (30 dB per ring, taking into account propagation, splitting, and coupling losses), as well as extinction ratio for the ring resonances (10–15 dB). One of the fabricated PICs was used in a preliminary experiment for detection of PCV2 virus. After the rings functionalization with specific antibodies and attaching a microfluidic layer on the PIC, positive detection of several virus concentrations was achieved, demonstrating the good performance and feasibility of the proposed detection technique.
